# Factors Affecting Extracellular Vesicles Based Drug Delivery Systems

**DOI:** 10.3390/molecules26061544

**Published:** 2021-03-11

**Authors:** Isha Gaurav, Abhimanyu Thakur, Ashok Iyaswamy, Xuehan Wang, Xiaoyu Chen, Zhijun Yang

**Affiliations:** 1 School of Chinese Medicine, Hong Kong Baptist University, Hong Kong, China; ishagaurav@life.hkbu.edu.hk (I.G.); ashokenviro@gmail.com (A.I.); 19424019@life.hkbu.edu.hk (X.W.); cxyu2016@hkbu.edu.hk (X.C.); 2 Centre for Regenerative Medicine and Health, Hong Kong Institute of Science and Innovation-CAS Limited, Hong Kong, China; abithakur1211@gmail.com; 3 Mr. & Mrs. Ko Chi-Ming Centre for Parkinson’s Disease Research, School of Chinese Medicine, Hong Kong Baptist University, Hong Kong, China; 4 Changshu Research Institute, Hong Kong Baptist University, Changshu Economic and Technological Development (CETD) Zone, Changshu 215500, Jiangsu Province, China

**Keywords:** extracellular vesicles, exosomes, drug delivery systems, targeted delivery

## Abstract

Extracellular vesicles (EVs) play major roles in intracellular communication and participate in several biological functions in both normal and pathological conditions. Surface modification of EVs via various ligands, such as proteins, peptides, or aptamers, offers great potential as a means to achieve targeted delivery of therapeutic cargo, i.e., in drug delivery systems (DDS). This review summarizes recent studies pertaining to the development of EV-based DDS and its advantages compared to conventional nano drug delivery systems (NDDS). First, we compare liposomes and exosomes in terms of their distinct benefits in DDS. Second, we analyze what to consider for achieving better isolation, yield, and characterization of EVs for DDS. Third, we summarize different methods for the modification of surface of EVs, followed by discussion about different origins of EVs and their role in developing DDS. Next, several major methods for encapsulating therapeutic cargos in EVs have been summarized. Finally, we discuss key challenges and pose important open questions which warrant further investigation to develop more effective EV-based DDS.

## 1. Introduction

**Figure 1:**
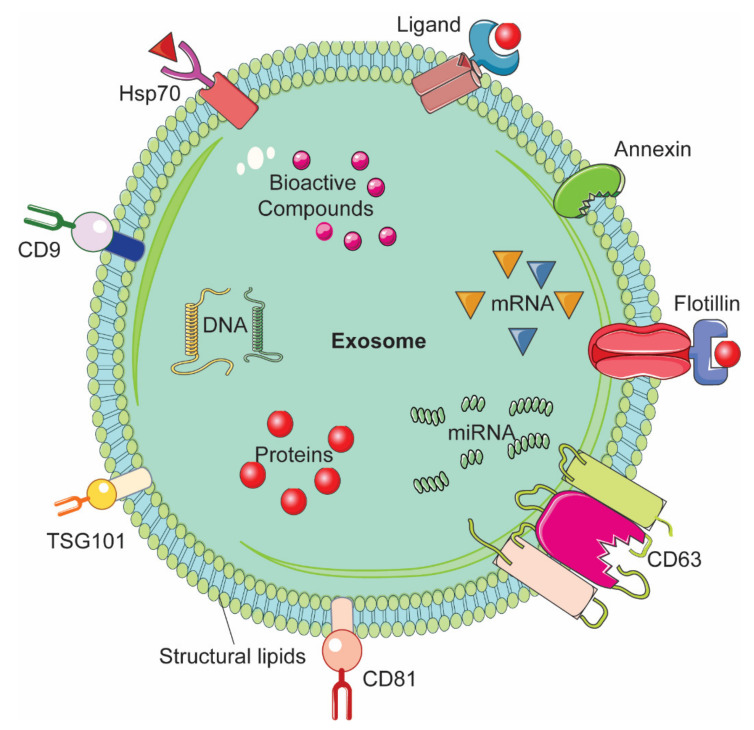
Representative structure of an exosome and its composition.

A drug delivery system (DDS) consists of various formulation which enable therapeutic substance to reach the desired site of action specifically without going to non-target sites [1]. In nano drug delivery systems (NDDS), different biodegradable and biocompatible materials with size approximately 10-100 nm are utilized as nanocarriers [2,3]. These nanocarriers can be either natural or synthetic polymers, lipids, and metals such as nanoparticles [1,2,3,4]. Although NDDS have been used with several drugs including anti-cancer drugs [5,6,7], very few have been approved for use in humans by the Food and Drug Administration [8]. Cytotoxicity and rapid clearance of most of the synthetic NDDS via the mononuclear phagocyte system or reticuloendothelial system have been major bottlenecks in their transition from bench to bedside in clinical setting [9,10]. Several approaches have been employed to modify the nanoparticles (NPs). One example is coating the NPs with polyethylene glycol (PEG); this enhanced circulation time but impeded interaction between the target cells or tissues and the NDDS, thereby interfering with their biodistribution [11,12,13]. Another approach is to look for natural DDS, which could be expected to yield higher therapeutic value owing to their better in vivo biocompatibility as compared to the synthetic NDDS [14,15,16]. Extracellular vesicles (EVs) are natural nanovesicles released from most cells and biofluids; they carry various cargo including nucleic acids, proteins, and lipids [17]. EVs have attracted tremendous attention in the context of NDDS due to their ability to facilitate intracellular communication and the transportation of cargo to the target recipient cells [18,19]. Based on their size range and biogenesis, EVs are categorized into three major types, namely exosomes, microvesicles (MVs), and apoptotic bodies (ABs) [20]. Exosomes are of endocytic origin. They have sizes in the range of 30–100 nm; structurally, exosomes are composed of a lipid bilayer carrying cargoes of different composition including functional proteins, DNA, mRNA, miRNA, and lncRNA (Figure 1) [21,22,23].

**Figure 2:**
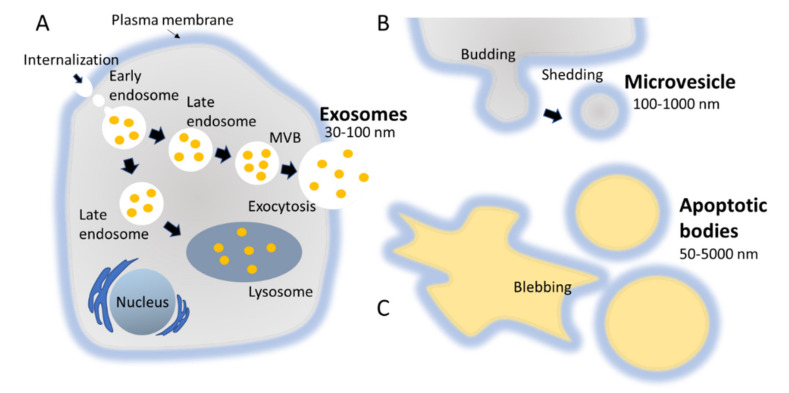
Biogenesis pathways followed by different types of EVs, namely exosomes, MVs, and ABs. (**A**) MVBs fuse with lysosomes and degrade or fuse with the plasma membrane and form exosomes which are released from cell into extracellular space. (**B**) MVs are a set of EVs with size in the range of 100–1000 nm; they are formed and released by budding off cell membrane. (**C**) ABs are in the size range of 50–5000 nm, and are released from cells undergoing apoptosis.

The biogenesis of exosomes takes place by inward budding of the plasma membrane that forms the endosome vesicle, and the multivesicular bodies (MVBs). MVBs fuse with lysosomes and degrade or fuse with the plasma membrane and form exosomes which are released from cell into extracellular space (Figure 2A) [24,25]. They are released from various cell types like red blood cells, platelets, lymphocytes, dendritic cells (DCs), epithelial cells, adipocytes, fibroblasts, neural cells, stem cells, and cancer cells [26]. They have also been found in various biofluids such as blood, plasma, urine, cerebrospinal fluid (CSF), milk, amniotic fluid, malignant ascites, saliva, and synovial fluid [27,28,29]. They play a major role in cell-to-cell communication in the signalling pathways of both physiological and pathological processes [30], and transferring molecules such as proteins and RNA from donor cells to recipient cells [27,31]. Various specific proteins are present on the surface of exosomes, such as tetraspanin proteins (CD9, CD63, and CD81) [31], lysosomal protein (Hsp70), tumor sensitive gene 101 (Tsg101), and fusion proteins (annexin, and flotillin) [32]. These proteins are associated with the endosomal pathway, and are characteristic of exosomes, distinguishing them from MVs and ABs. MVs are another set of EVs with size in the range of 100–1000 nm; they are formed and released by budding off cell membrane [33]. In contrast, ABs are in the size range of 50–5000 nm, and are released from cells undergoing apoptosis [34] as shown in Figure 2B, C. Some scientists have subdivided the category of EV into subtypes, partially based on size, marker and biogenesis [35,36]; in this review the term EV refers to the general category, unless we specifically refer to a subgroup.

A diverse range of studies have been carried out exploring the application of EVs in drug delivery. It has been found that miRNA and protein can be loaded on EVs and delivered to t tumor cells [37,38]. EVs also show the capability to inhibit tumor growth by delivering chemical drugs [39]. Interestingly, EVs can avoid phagocytosis by macrophages and prolong the half-life of chemical drugs in comparison to artificial NDDS, and are considered as the natural carrier of chemical drugs to improve the efficiency of biological drug delivery [40,41,42]. Previous review articles have covered various aspects of EVs in NDDS. This review updates those summaries with an in-depth discussion of the latest methods for surface modification of EVs, the importance of cell origin, and the importance of loading efficiency in EV-based targeted drug delivery systems. Here we shed light on the structural similarity between liposome and EVs, and their different roles in targeted delivery.

## 2. Exosomes and Liposomes: How Similar Are They?

**Figure 3:**
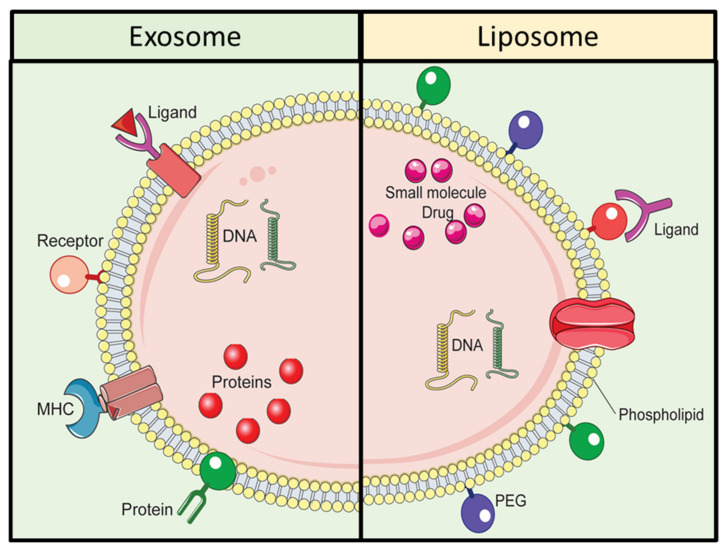
Comparative schematic illustration of exosomes and liposomes.

Among various synthetic NPs, liposomes share some similarities with exosomes, one of the major types of EVs. Figure 3 is a representative depiction of exosomes and liposomes in the context of their structure and composition. Exosomes and liposomes are composed of lipid bilayer membrane structure with various dissimilarities. The membrane of exosomes contains various proteins such as tetraspanins, and lipids to enable effective uptake and targeting. Liposomes can be produced via diverse amalgamation of lipids, and their circulation time can be extended by using surface modification with polyethylene glycol. Although liposomes are produced synthetically, their lipid bilayer structure is like that of exosomes. The formulation of liposomes allows the loading of different types of cargo., For example hydrophilic molecules including DNA, RNA, and siRNA can be encapsulated inside the aqueous core of liposomes, and hydrophobic compounds including peptides, proteins, and antibodies can be encapsulated in the lipid bilayer of liposomes [43]. Liposomes can be produced via various methods, such as sonication, membrane extrusion, freeze-thaw, and micro-emulsification [44,45]; however production can be challenging owing to the need for various chemical treatments. In addition, there are multiple steps involved in the modification of the lipid bilayer of the liposomes by using ligands, proteins and other functional elements [46,47]. Surface modification is a crucial factor in achieving targeted DDS. Notably, the limitations due to the tedious and time-consuming steps in the functionalization of the lipid bilayer of liposomes can be overcome via the application of EV-based DDS. Interestingly, exosomes share similar physicochemical characteristics with liposome; however, one important difference is that exosomes, unlike liposomes, are released naturally, as mentioned earlier. Importantly, exosomes can efficiently deliver exogenous hydrophilic molecules, and their intrinsic biochemical similarities with that of their originating parent cells [48] enable them to perform better in DDS, as compared to liposomes [49,50,51]. Table 1 enlists a comparison between exosomes- and liposomes for application in DDS.

Recently, liposome- and exosome-EVs have been proposed as hybrid nanocarriers in advanced DDS. For example, Sato YT et al. developed a hybrid exosome via the fusion of the membranes of exosomes with liposomes employing the freeze-thaw technique. Particularly, exosomes expressing specific membrane proteins were isolated from genetically modified cells, then fused with several liposomes. Interestingly, uptake assay revealed that the interactions between exosomes and the recipient cells can be modified via the altering membrane lipid composition or characteristics of exogenous lipids in hybrid exosomes [52].

## 3. How Crucial Are Isolation, Yield, and Characterization of Evs for Delivery Purposes?

The isolation of EVs with good quality and high yield from culture-conditioned medium or from biofluids is important for developing EV-based DDS. The isolation of different types of EVs such as exosomes, MVs, and ABs can be carried out based on different parameters including size, density, surface markers, and molecular composition [63]. The qualities and the yield of EVs mostly depend on the type of method employed for their isolation and purification. The most conventional method for isolating EVs is differential ultracentrifugation (DUC), which involves multiple centrifugation steps to eliminate different particles, such as cell debris, at various steps, eventually yielding exosomes in the form of pellets [64]. Although this method is widely used in many labs, there are several limitations and disadvantages. First, it requires heavy instruments, much time, much patience due to the tedious nature of the procedure, and large volumes of sample [52]. Second, there is a probability of loss of and damage to the EVs due to the repeated centrifugation steps.

Various other strategies have been developed including density gradient centrifugation, immunomagnetic bead-based extraction, chromatography, ultrafiltration, and microfluidic device. In addition, various commercial precipitation kits are available such as ExoQuick (from System Biosciences), Total Exosome Isolation Reagent kit (from Thermofisher), and ExoSpin Exosome Purification Kit (Cell Guidance Systems). The advantages and disadvantages of most of these methods have been extensively reviewed elsewhere [53]. Regardless of the numerous available methods, there is no agreement with respect to the best procedure for the isolation of different types of EVs, delivering significant yields with consistent quality [65].

Another important aspect that should be considered while isolating EVs for developing DDS is the yield of EVs. This factor is particularly important in terms of the cost of production. Various advanced methods have been reported recently for isolating EVs with better yield. For example, Liu et al. developed an exosome total isolation chip (ExoTIC) for the size-dependent isolation of EVs. This method was found to be simple, convenient to use, and modular; it produced a high yield of EVs with enhanced purity from biofluids. Remarkably, ExoTIC reportedly achieved a yield of EVs approximately 4-1000-fold higher as compared to the conventional techniques including DUC [66]. Two other strategies have been explored for enhancing yield. One is stimulation of donor cells with liposomes. One study revealed that incubation of tumor cells with neutral and cationic-bare liposomes enhanced the release of EVs, suggesting that, depending on physicochemical properties of liposomes, they can either behave as stimulant or depressant on the release of EVs from tumor cells [67]. The second strategy is the application of stimulating agents, for example in one study, monensin antibiotic has been shown to stimulate the release of EVs in a calcium-dependent manner [68,69].

The source of EVs also plays a crucial role in determining the yield of EVs. For example, mesenchymal stem cells (MSCs) have been reported to be a popular source of EVs for developing into DDS [70]. Importantly, MSCs are easily available and can be produced on a large scale. MSCs release the highest number of EVs as compared to other cell types [71]. In addition, the MSC-derived EVs are reported to show better selective targeting of locations of inflammation and injury. MSC-derived EVs migrate towards a tumor; this propensity can be utilized in developing potential cancer treatments and in regenerative medicine [72]. The origin of EVs has been reported to determine the ability of cells to target and transfer therapeutic cargo to specific recipient cells [73]. In summary, the isolation of EVs can be optimized for higher yield by considering the parameters of isolation technique and EV source. The precise characterization of EVs or modified EVs is important in any EV-related study. It is critical to be able to establish whether isolated vesicles are indeed EVs, and/or to validate the surface modification or the biochemical constituents of isolated EVs [74,75]. Various methods have been extensively employed by numerous scientists to study different parameters of EVs. These methods include: measuring size distribution of EVs via nano tracking analyzer (NTA), determining the morphology and size via transmission electron microscopy (TEM), and examination of exosomal proteins by immunogold-EM, Western blotting, and enzyme linked immunosorbent assay (ELISA) [76]. These methods have already been reviewed in detail elsewhere [77,78]. Recently, several new approaches have been employed by different research groups. One example is the application of surface plasmon resonance (SPR) for the study of surface markers. This method is useful for characterizing the subtypes of EVs based on different proteins on their surface [79,80]. SPR technology has also been utilized for measuring the concentration of EVs in solution; this is useful in the study of EV-based DDS [81]. Despite being an advanced, label-free tool, the SPR method has two serious drawbacks, namely the use of various chemicals in the immobilization of antibody against target antigen to be detected on the surface of EVs, and the need to enhance sensitivity [82]. Another technique, atomic force microscopy (AFM), entails the visualization of the three-dimension surface structure of EVs and the study of their nano-mechanical characteristics [83]. Several other analytical tools are being employed by different research groups to characterize and validate various types of surface of modification of EVs, including Fourier Transform Infrared Spectroscopy (FTIR), and Raman spectroscopy [84]. Several research groups have employed an integrated approach, combining different techniques to achieve better characterization of EVs [85]. Each method, each tool has its own advantages and disadvantages; the selection of which tool to use mostly depends on the type of study to be done.

## 4. Surface Modification of Evs for Targeted Delivery

Various strategies have been explored for the modification of EVs for targeted delivery such as click chemistry, and genetic engineering. Various targeting molecules including aptamer- and peptide anchorage to the surface of EVs have been employed, which will be described below.

### 4.1. Click Chemistry

In this method, an alkyne group is attached to the surface of isolated EVs through 1-ethyl-3-(3-dimethylaminopropyl) carbodiimide-N-hydroxysuccinimide (EDC-NHS) condensation reaction. The alkyne group further covalently attaches to the azido group of the targeting structure (on the surface of EVs) in the presence of copper. This approach offers various advantages. First, it is resistant towards fluctuation in temperature and pressure. Second, the reaction of click chemistry can take place in an aqueous buffer as well as organic solvent, without consuming much time. Thirdly, and importantly, the conjugation via click chemistry does not influence the size of EVs nor their uptake by recipient cells [86]. Various groups have employed this approach for the surface modification of EVs. For example Jia et al. loaded superparamagnetic iron oxide nanoparticles (SPIONs) and curcumin (Cur) into EVs and conjugated their membrane with neuropilin-1-targeted peptide (RGERPPR, RGE) via click chemistry to achieve glioma-specific targeting [87]. Liang et al. produced azido- modified EVs from MDA-MB-231 cells by changing their glycosylation pathway, and the azido group on EVs further reacted with azadibenzylcyclooctyne (ADIBO) fluorescent dyes via click chemistry, which were useful for in vivo tracking [88]. Although the click chemistry method has been widely explored for surface modification of EVs, its use is limited by the non-specific interaction between the targeting structure and the surface of EVs [89]. In addition, some scientists believe over-modification of proteins on the surface of EVs with alkyne groups may inhibit the function of those exosomal proteins [86].

### 4.2. Genetic Engineering

Various proteins are present in the membrane of EVs such as CD9, CD63, CD81, and Lamp, which can be conjugated with target ligands and can facilitate localized delivery of EVs [90]. Another crucial approach is engineering the donor cells producing the EVs by using plasmid vectors. For example, Kim et al. performed co-transfection of pcDNA-cardiac targeting peptide (CTP)-Lamp2b in HEK293 cells for producing EVs targeting cardiac cells [91]. Tian et al. transfected immature DCs with pEGFP-C1-iRGD-Lamp-2b plasmids for producing iRGD(CRGDKGPDC)-Lamp-2b positive EVs to target breast cancer cells in vitro and in vivo [92]. Modification of the surface of EVs with peptides has also been explored owing to their small size, high binding affinity, target specificity, low immunogenicity, and low toxicity. Moreover, peptides re conveniently tunable and can be synthesized based on the information about their targeting ligand and screening of library of peptides [93]. Leonard et al. conjugated targeting peptides (having glycosylation sequence GNSTM) with EVs to suppress peptide loss and enhance the delivery of EVs to neuroblastoma cells, suggesting that glycosylation does not ablate the interaction between peptide and target [94].

## 5. Targeting Molecules-Based Engineering of E*V*s

### 5.1. Aptamer-Based Surface Modification of EVs

Aptamers are short, single-stranded DNA, RNA, or xeno nucleic acid (XNA), which can be produced with high specificity and affinity towards desired targets by PCR-based in vitro selection;, this approach is referred to as systematic evolution of ligands by exponential enrichment (SELEX) [95,96,97]. Aptamers have also been employed in surface modification of exosomes for targeted delivery. For example, Wan et al. developed aptamer grafted EVs loaded with paclitaxel for targeting breast cancer in vivo. To accomplish this, nucleolin-targeting aptamer AS1411 was attached to cholesterol-polyethylene glycol covalently. The resultant compound was anchored on the membrane of mouse DCs, followed by extrusion of cells by passing them through micro constrictions to get EVs and loading with paclitaxel via sonication method [98]. In another study, Pi et al. modified EVs by displaying an aptamer, which could bind to prostate-specific membrane antigen and loaded the modified EVs with survivin siRNA. This aptamer decorated EV loaded with surviving siRNA effectively inhibited the growth of a prostate cancer xenograft. Moreover, the same EV-based delivery system displaying EGFR aptamer could inhibit orthotropic breast cancer [99]. Zou et al. developed an aptamer-functionalized exosome for cell type-specific delivery by employing diacyl lipid- sgc8 aptamer conjugates as the targeting ligands. The aptamer sgc8 could specifically recognize membrane-bound protein tyrosine kinase 7 (PTK7), suggesting its applicability as an important theranostic platform [100].

### 5.2. Peptide Anchoring on EVs

Anchoring peptide on the surface of EVs is a successful strategy that has been employed by many research groups for achieving targeted delivery. For example, Alvarez-Erviti et al. engineered DCs for expressing Lamp2b, an exosomal membrane protein, conjugated with neuron-specific RVG peptide. The exosomes were encapsulated with siRNA via electroporation. The intravenous injection of RVG-targeted exosomes could deliver siRNA specifically to brain cells including neurons, microglia, and oligodendrocytes, and showed specific gene knockdown [101]. Zhang et al. performed conjugation of c(RGDyK) peptide on EVs isolated from mesenchymal stromal cell (MSC), followed by loading with cholesterol-modified miR-210. This delivery system was found to be effective for the treatment of ischemic stroke [102]. Another research led by Zhan et al. engineered blood EVs via binding of magnetic molecules and endosomolytic peptides, L17E on the surface of EVs, followed by co-embedding doxorubicin (Dox) and cholesterol-modified miRNA21 inhibitor (miR-21i). Importantly, the surface engineering of blood EVs could enhance the tumor accumulation and increased capacity to escape endosomes, thereby leading to specific and efficient delivery of loaded cargoes to tumor cells in vitro and in vivo [103].

## 6. How Crucial Is the Origin of Exosomes for Drug Delivery?

**Figure 4:**
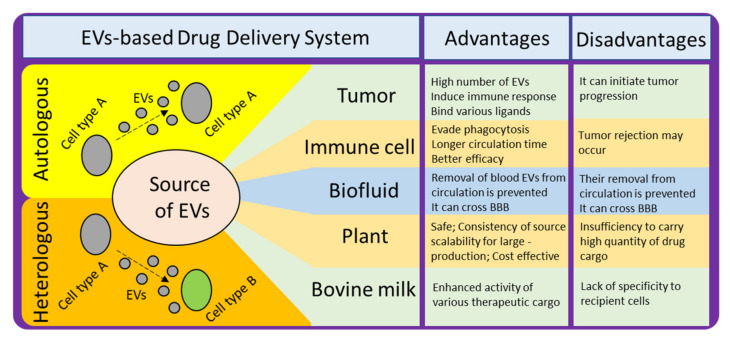
Different sources of EVs for developing EV-based DDS, their advantages, and disadvantages.

There are various sources of EVs which can be employed for developing DDS (Figure 4). To achieve the desired effect and to avoid any potential harmful effects, it is necessary to understand the pros and cons of selecting any particular source. For example, the proportion of lipid to surface protein in an EV differs, depending on the source [104]. This proportion can affect certain properties that are crucial for effective delivery, and thus should be considered when selecting a source. It has also been found that the proportion of certain lipids is enhanced in exosomes as compared to the amount of lipids in their donor cells, such as sphingolipid, phosphatidylserine, phosphatidylinositol, and cholesterol. These lipids facilitate in enhancing the rigidity of exosomal membrane [105]. Another factor to consider is the relationship between surface proteins and target cells. Some sources produce EVs with surface proteins that are detrimental to the recipient target cells. A third factor is biocompatibility; and finally yield should also be taken into consideration while making selection of donor source cells for isolation of EVs for developing into EVs-based DDS.

### 6.1. Choice between Autologous and Heterologous EVs

The choice between autologous and heterologous (also referred as allogenic) EVs for developing into DDS is one of the crucial factors for effective delivery. It has been found that the uptake of autologous EVs and the uptake of heterologous EVs by target recipient cells differ distinctly. As the compositions of EVs have been reported to mimic their parent cells, the selection of heterologous EVs may induce an immune response in the target recipient cells. Therefore, theoretically, autologous EVs may be more suitable for therapeutic purposes [72]. In practice, however, heterologous EVs from MSCs have been found to be safe and reliable for therapeutic purposes. Still, we shouldn’t forget about autologous EVs, for example, pathological tissues are generally considered waste; however, if the EVs from these tissues could be isolated and their disease-causing cargo removed, these EVs could be valuable DDs [106]. Lessi et al. demonstrated that human primary macrophage-derived EVs could deliver drugs efficiently, [107], suggesting that autologous EVs derived from peripheral blood-derived primary monocytes could be suitable as theranostic agents. The safety profile of these EVs needs to be assessed before developing them into DDS; however, some evidence indicates they are safer than EVs derived from plasma [108].

### 6.2. Tumor-Derived EVs

Tumor-derived EVs (TEVs) have been employed by many research groups for drug delivery [109,110]. TEVs have several advantages compared to other delivery carriers. For example, tumor cells release significantly high numbers of EVs [111], suggesting their suitability for studies requiring large amounts of EVs. In addition, TEVs carry MHC class-I molecules and antigens specific to the originating tumor cells. Moreover, TEVs can induce immune response against cancer cells by delivering antigens to DCs [112]. Interestingly, the tetraspanin proteins, common markers for exosomes, have been found to bind with various ligands in a diverse range of tissues, suggesting their suitability for targeted delivery [113]. TEVs from melanoma patients have been found to increase the release of myeloid-derived suppressor cells (MDSCs), crucial for avoiding immune recognition [114,115]. It is striking that although TEVs have been shown to have potential for targeted delivery, there is also a chance that they can initiate tumor progression due to various of their constituents, such as urokinase plasminogen activator, which can promote cancer cell invasion, and adhesion modulators like vimentin, and annexin A1 [116]. Therefore, again, selection of appropriate source for isolating EVs is a crucial factor for developing successful and effective EV-based targeted DDS.

### 6.3. Immune Cell-Derived EVs

Another important source from which EVs can be isolated are immune cells. For example cells like macrophages and monocytes have gained attention for EV-based immunotherapy [117,118]. Immune cell derived EVs (IEVs) can evade phagocytosis, a clearance mechanism, which is a major limitation for most of the other types of EVs. Therefore, IEVs possess longer circulation time and better efficacy [119]. Importantly, the DC-derived EVs (DCEVs) seem to have a great potential as various clinical studies have demonstrated their effectiveness on different cancers. DCEVs play a major role as intercellular communicators in adaptive immunity for modulation of immune responses. Therefore, most of the researches related to DCEVs are about immunotherapy of cancer leading to clinical advantage [120,121]. Notably, in a Phase-I clinical trial, Escudier et al. reported the feasibility and safety of administering DCEVs pulsed with MAGE 3 peptides for immunization in melanoma patients under stage- III/IV [118]. DCEVs have also been found to promote tumor rejection via transporting peptide-MHC complexes from DCs (exposed to an antigen) to other DCs (not exposed to same antigen) [117,122,123].

### 6.4. Biofluid-Derived EVs

EVs derived from biofluids such as plasma [124], and ascites [125], have shown potential as delivery carriers. Biofluid-derived EVs have several advantages as delivery carriers. For example, unlike cell culture-derived EVs, plasma-derived EVs are enriched with lyso-phospholipids and do not contain phosphatidylserine (PS). The absence of PS on the surface of plasma-derived EVs prevent their removal from circulation [124,126]. In addition, plasma-derived EVs can cross the blood-brain barrier (BBB), which suggests their applicability for brain delivery [124]. Ascites-derived EVs along with granulocyte–macrophage colony-stimulating factor have been found to be safe and effective for immunotherapy of colorectal cancer [125]. It has also been reported that human peripheral blood-derived EVs loaded with miRNA have potential for treating cardiac diseases [127]. Another study showed that EVs in peripheral blood can be important mediators of lung injury via exosomal shuttling of miR-155 [128]. Blood EVs have been shown to be crucial for targeting brain disease. For example, dopamine-loaded blood EVs can be used as delivery platform in treating Parkinson’s disease and other central nervous system-related disorders [129]. Urine- and saliva-derived EVs have not been much explored for their therapeutic potential as delivery carriers; however, they have been extensively studied for developing biomarker of different diseases including cancer [130,131]. Conclusively, biofluids such as blood and ascites are great sources of EVs for developing novel DDS.

### 6.5. Plant and Bovine Milk-Derived EVs

Due to safety concerns related to TEVs and IEVs, scientists have explored the applicability of plant-derived EVs (PEVs) as DDS, such as grape-derived EVs [132] or bovine milk-derived EVs (BMEVs) [133]. There are several advantages of using PEVs as DDS, including better safety, consistency of source, scalability for large production, and relative cost effectiveness [132]. Several research groups have isolated EVs from different plants or food sources and showed a diverse range of applications [134]. For example Ju et al. demonstrated that grape- derived EVs are useful in protecting intestinal damage in mice via facilitating growth and differentiation of intestinal stem cells [132]. Subsequently, Wang et al. demonstrated that modification of grapefruit derived EVs improved their ability to target tumors and loaded those EVs with doxorubicin and curcumin. Interestingly, those EVs were found to be effective against inflammation in vivo [135]. Bovine milk is another important source of EVs for DDS. Mungala et al. demonstrated the enhanced activity of various therapeutic cargo loaded BMEVs against lung cancer in vitro and *in vivo*. They further showed that modification of BMEVs with folate could enhance tumor-targeting ability as compared to the free drug [133]. Recently, because many clinical trials for the treatment of Alzheimer’s disease (AD) using synthetic drugs have failed [136,137,138], scientists are trying to develop targeted delivery using EVs and developing precision medicine-loaded EVs for the treatment of AD [139,140]. Plant-derived traditional medicine have been studied in preclinical models of AD [141,142,143]. Targeted delivery of plant-derived bioactive components using EVs could be more effective for the treatment of AD.

## 7. How Does Loading Efficiency Play a Crucial Role in EV-Based DDS?

Loading therapeutic cargo into EVs is one of the crucial parts in the process of developing EV-based DDS. The high loading efficiency of EVs ensures better bioavailability of the cargo when it reaches the target site. The major factors that need consideration while loading any cargo onto EVs are: how better encapsulation or loading efficiency can be achieved, how the structural integrity of EVs can be maintained, and how the functional properties of the therapeutic cargo can be maintained.

**Figure 5:**
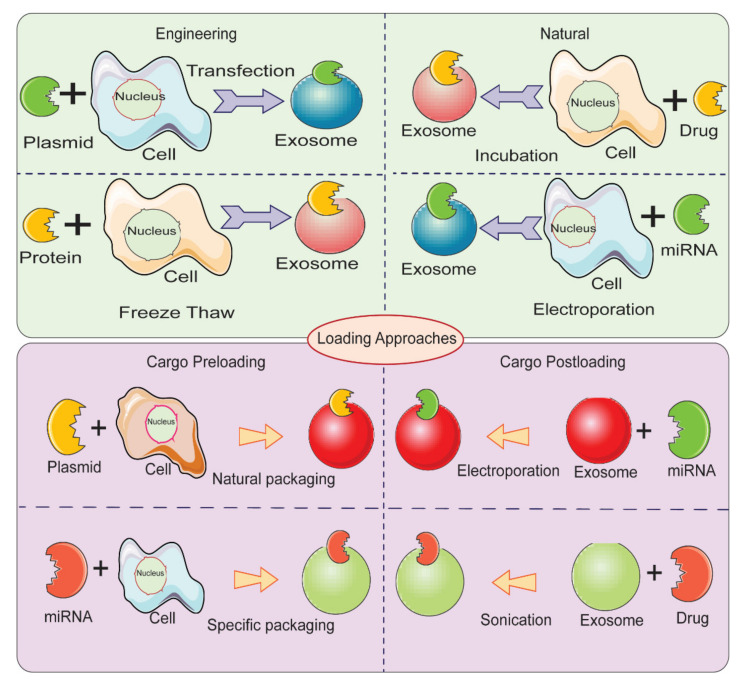
Different methods for loading therapeutic cargos in EVs for developing EV-based delivery systems.

Therapeutic cargo such as proteins, drugs, or small nucleic acids such as miRNA can be loaded in two different ways. First, the therapeutic cargo can be incorporated into donor cells, followed by isolation of EVs; this is referred to as in vitro loading [144]. Second, the therapeutic cargo can be loaded after isolation of EVs via various methods including incubation, sonication, electroporation, extrusion, permeabilization, or the freeze-thaw method; this is referred to as ex vivo loading (Figure 5) [119]. In the simple incubation method, EVs are incubated with drugs, and the drugs enter EVs via diffusion due to the concentration gradient. Incubation is found to be suitable for loading hydrophobic drugs as they interact with lipid layers of EVs’ membranes [119,145]. One disadvantage of simple incubation is low loading efficiency. Another method of loading is incubation of drugs with donor cells, followed by isolation of EVs [144]. Table 2 summarizes different loading methods with their advantages and disadvantages. The physicochemical properties of the therapeutic cargo partially determine what method is employed for their encapsulation in EVs. For example hydrophobic drugs such as curcumin can be loaded within the inner layers of fatty acid via incubation, whereas hydrophilic molecules including siRNA, miRNA can be loaded by forming transient pores on the membrane of EVs via methods like electroporation [16,101,146].

Recently, membrane permeabilization of EVs has been found to be a promising method for enhancing the loading efficiency of EVs. Saponin has been shown to be particularly effective in enhancing the loading of different cargos in EVs from various sources. Being a surfactant, saponin is able for form a complex with cholesterol in the membranes of cells and create pores, thereby facilitating permeabilization [147]. Haney et al. demonstrated that loading efficiency of catalase into exosomes can be enhanced via incubation with saponin, as compared with simple incubation technique. Interestingly, the activity of catalase was not affected by the saponin [119]. Another recent research showed that passing saponin through the microfluidic channels enhances loading of doxorubicin in glioma stem cell-derived exosomes as compared with other conventional methods. The authors reported two different microfluidic channels; one linear. and another sigmoid, which suggests that designing advanced microfluidic channels along with using permeabilizing agent may have a synergistic effect to achieve augmented efficiency of loading cargoes in EVs [16,57]. Fuhrmann et al. showed that incubation of a small hydrophilic molecule, porphyrin, with saponin could enhance the loading efficiency as compared with a passive loading technique excluding saponin [148]. Nevertheless, there are some concerns associated with the use of saponin for in vivo purposes because of its hemolytic activity [147].

## 8. Conclusions and Future Directions

The arrival of EVs has created a paradigm shift in the development of NDDS. The use of EVs shows the potential to overcome most of the drawbacks of conventional NDDS such as metal based nanoparticle DDS, and liposomes. EV-based DDS have shown remarkable advantages including better biocompatibility, the homing effect, and convenient methods for modification of their surfaces for targeted delivery. Although exosomes have several advantages as DDS, however the functional delivery of EVs-based DDS is mostly challenged owing to lack of effective and reproducible loading methods [50]. Nevertheless, concerns related to indigenous functional cargos present in EVs arise owing to their potential to initiate various unwelcome events such as immunosuppression or promotion of tumorigenesis [154,155]. The source of an EV determines many of its properties. Therefore, it is crucial to determine which sources are safe and reliable for the isolation of EVs. To achieve target-specific DDS, EVs can be functionalized with exogenous materials including ligands, peptides, and proteins. Various methods have been employed for accomplishing augmented loading efficiency of cargos in EVs, and this aspect needs further investigation to improve encapsulation of therapeutic cargo in EVs. Although many studies have been carried out exploring the potential of EV-based DDS, many challenges remain, particularly related to isolation and characterization and, ultimately, to their translation into clinical therapeutics. Furthermore, there is a demand to improve the amount of EVs that can be extracted from cell-conditioned medium; clinical needs will far exceed what can be produced in an experimental laboratory. Certainly, this issue can be overcome, but the cost of producing EVs must be controlled such that EV-based DDS are affordable. Developing target and site specific EV-based DDS is an important aspect for effective delivery to site of action without damage to normal cells in the vicinity. Considering all the above-mentioned discussion, the use of EV as a nanocarrier has profound values for the development of effective DDS and hold a great promise for clinical translation.

## Figures and Tables

**Table 1:** Comparison between exosomes and liposomes for DDS application.

.	Exosomes	Ref.	Liposomes	Ref.
**General preparation method**	The commonly used methods are traditional ultracentrifugation, density gradient separation, immunomagnetic beads, ultrafiltration, and commercial reagent-based isolation.	[53,54]	Most of the methods involve four basic steps: (a) Drying down lipids from organic solvent. (b) Dispersing the lipid in aqueous media. (c) Purifying the resultant liposome. (d) Analyzing the final product.	[55]
**Yields**	Varies depending on isolation method; commercial reagent such as Invitrogen kit could between 200 and 300 µg exosomal protein amount, whereas immunomagnetic beads-based isolation method produces least amount of exosomes.	[54]	Up to 100% by freeze-drying	[56]
**Loading efficiency**	Low (5–50% depending on methods)	[16,57,58]	High (30–90% depending on methods)	[59]
**Advantages**	High organotropism effect; immune-compatible if isolated from autologous donor cells.	[60]	Increased efficacy and therapeutic index of drug; Increased stability via encapsulation; non-toxic, flexible, biocompatible, completely biodegradable, and non-immunogenic for systemic and non-systemic administrations; reduce the toxicity of the encapsulated agent; reduce the exposure of sensitive tissues to toxic drugs; Site avoidance effect; Flexibility to couple with site-specific ligands to achieve active targeting	[55]
**Disadvantages**	Rapid clearance from blood after in vivo administration; Low drug loading efficiency; Unavailability of universally accepted standard manufacturing process.	[60]	Low solubility; Short half-life; Sometimes phospholipid undergoes oxidation and hydrolysis-like reaction; Leakage and fusion of encapsulated drug/molecules; Production cost is high; a few stability issue	[55]
**Clinical progress**	Engineered exosomes in preclinical development: Exosome–lipid nanoparticle hybrid loaded with RNA or DNA (Anjarium Bioscience); Cow’s milk exosomes loaded with RNA or DNA (PureTech) Engineered exosomes in phase-I clinical trials: Cell culture exosomes loaded with KRAS-G12D siRNA (MD Anderson Cancer Center), cell culture exosomes loaded with IL-12 or STRING agonist (Codiak Biosciences)	[61]	Already available as clinical products, for example Doxil^®^, Ambisome^®^, DepoDur™.	[62]

**Table 2:** Different techniques for loading cargos in EVs with advantages and disadvantages.

Loading Methods	Steps Involved	Advantages	Disadvantages	Ref.
Electroporation	Phospholipid bilayer of EVs are disorganized by an electric field, creating pores in the membrane which allow the passage of drug to vesicle.	Loading with large molecules is possible	Disrupts integrity of EVs; Low loading efficiency	[101,149,150,151]
Sonication	Exosomes derived from donor cells are mixed with drug and sonicated through probe sonicator which permits the drug to flow into exosome	Increased loading efficiency; applicable for small RNAs	Potential deformation of membrane of EVs; Not efficient for hydrophobic drugs.	[60,152]
Extrusion	Exosomes are mixed with drug and loaded into syringe-based lipid extruder and extruded through membrane with 100–400 nm pore size at controlled temperature.	High drug loading efficiency	Potential deformation of membrane.	[60]
Freeze/Thaw Method	Exosome are mixed with drug and incubated, subsequently frozen at −80 °C or in liquid nitrogen and are thawed at room temperature.	Medium loading;Fusion of membranespossible	Exosomes may aggregate; Low loading efficiency	[60]
Saponin-Assisted Loading	Saponin is incubated with exosomes to generate pores in their membrane by interacting with cholesterol which leads to increased membrane permeability	High drug loading compared to the other methods used in early reports	Generates pores in exosomes; Saponin can cause haemolysis; Toxicity concerns; Saponin concentration control & washing required	[119,147,148]
Dialysis	Exosomes mixed with drug are dialyzed by stirring to obtain drug loaded exosome.	Promotes loading efficiency	Poor cellular uptake; No substantial impact on photodynamic effect	[153]

## Data Availability

Not applicable.
